# Worker Illness Related to Newly Marketed Pesticides — Douglas County, Washington, 2014

**Published:** 2015-01-23

**Authors:** Geoffrey M. Calvert, Luis Rodriguez, Joanne Bonnar Prado

**Affiliations:** 1 Division of Surveillance, Hazard Evaluations, and Field Studies, National Institute for Occupational Safety and Health, CDC; 2 Washington State Department of Health

On April 10, 2014 the Washington State Department of Agriculture (WSDA) was notified by a local newspaper of a suspected pesticide poisoning incident in Douglas County involving pesticides not previously reported in the published literature to be associated with human illness. On that same day, WSDA notified the Washington State Department of Health, which investigated this incident by conducting a site visit, reviewing medical and applicator records, and interviewing affected farmworkers, pesticide applicators, and the farmworkers’ employer. In addition, on April 11, WSDA collected swab, foliage, and clothing samples and tested them for residues of pyridaben,* novaluron,† and triflumizole.§ In this incident, all 20 farmworkers working in a cherry orchard became ill from off-target drift of a pesticide mixture that was being applied to a neighboring pear orchard. Sixteen sought medical treatment for neurologic, gastrointestinal, ocular, and respiratory symptoms. This event highlights the need for greater efforts to prevent off-target drift exposures and promote awareness about the toxicity of some recently marketed pesticides. Incidents such as this could be prevented if farm managers planning pesticide applications notify their neighbors of their plans.

On April 8, 2014, two pesticide applicators were driving tractor-pulled airblast sprayers to apply a mixture of pesticides to prevent psylla infestations in a pear orchard.¶ At about 1:30 pm the tractors approached the end of the orchard, which abuts a cherry orchard. In the cherry orchard, 20 Hispanic farmworkers (19 women and one man) were tying the branches of cherry trees to trellises to improve fruit yields. Median age of the farmworkers was 33 years (range: 25–63 years). The workers were dispersed, and their distance from the edge of the pear orchard ranged from 30 to >350 feet (9 to >107 meters). The farmworkers and applicators disagree regarding when the applicators first observed the farmworkers and when the application ceased. The pesticide mixture included novaluron, pyridaben, and triflumizole, along with mineral oil,** boron (a micronutrient), and phosphoric acid (an acidifier, defoaming agent, and fertilizer).†† The farmworkers had not been notified of the pear orchard pesticide application before starting work in the cherry orchard.

All 20 cherry orchard workers reported that they began feeling ill within minutes of exposure to the drifting pesticides. The crew leader called 9-1-1. All of the workers reported two or more symptoms consistent with those caused by the pesticides applied to the pear orchard (1). Emergency medical services personnel decontaminated five workers at the orchard and transported them to an emergency department, where they were treated for their symptoms. A total of 16 workers eventually sought medical care. Six workers had moderate-severity illness, and the remaining 14 workers had low-severity illness.§§ The most commonly reported symptoms were neurologic (100%) (e.g., headache and paresthesias), gastrointestinal (95%) (e.g., nausea), ocular (85%) (e.g., eye pain/irritation), and respiratory (80%) (e.g., upper respiratory irritation and dyspnea) (Table). Of the eight workers who were contacted at least 2 weeks after the incident, six (75%) had symptoms that persisted for at least 2 weeks. The two applicators were wearing complete personal protective equipment (including air-purifying respirators and chemical-resistant headgear) and reported no symptoms.

Several of the samples collected by WSDA for pesticide residue analysis tested positive, including two clothing samples from farmworkers that tested positive for triflumizole. Both of these workers were working within 50 feet (15 meters) of the pesticide application. Residues of all three pesticides were found on cherry foliage. Residues of novaluron and pyridaben were found on the portable toilet used by the farmworkers (located at the boundary between the two orchards) and on the grass in the cherry orchard.

WSDA obtained wind speed and direction data from applicator and meteorologic records. Wind speed, measured hours before the incident by the applicators at the pear orchard using a handheld anemometer and documented in the application record, was low at 0–4 mph (0–6 kph), but the wind direction was variable. When the application began at 7:00 am, the wind direction was away from the cherry orchard, but at the time of the incident the winds were blowing in a circular pattern up to 18 mph (29 kph), and this is thought to have contributed to the incident.

## Discussion

This report highlights at least three potential occupational hazards in agriculture: off-target pesticide drift, toxicity of some recently marketed pesticides, and a gap in worker notification requirements. In this incident, off-target drift of a pesticide mixture was determined to be the cause of symptoms in 20 farmworkers. This finding is substantiated by the short distance between the site of pesticide application and the farmworkers location; the detection of pesticide residues on samples collected in the cherry orchard and on the worker’s clothing; the sudden onset of symptoms coinciding with the application; and symptoms that were consistent with those caused by the pesticides applied to the pear orchard. Off-target drift has previously been documented as the most common root cause of acute pesticide-related illness among farmworkers (2).

In the spring, pesticides are often applied to pear trees to prevent psylla infestations. Psylla can accumulate on leaves and fruit, reducing the plant’s photosynthetic capacity and producing deformed fruit with reduced commercial value. Because pests develop resistance to pesticides, there is a continual need to develop novel pesticides that attack different pest vulnerabilities. This is the first published report of illnesses associated with exposure to three recently introduced pesticides: pyridaben, novaluron, and triflumizole. The products applied to the pear orchard that contained pyridaben and novaluron were both toxicity category II pesticide products. Pyridaben is an insecticide and miticide that acts by inhibiting mitochondrial complex I electron transport. It was first approved to be sold in the United States in 1994. The product label for pyridaben warns that it can be fatal if inhaled and that pesticide applicators and handlers are required to use extensive personal protective equipment, including air-purifying respirators (3). It also can cause moderate eye irritation. Novaluron is an insect growth regulator that acts by inhibiting chitin synthesis. It was initially registered for sale in the United States in 2001. It is reported to cause substantial but temporary eye injury (4). Triflumizole is an imidazole fungicide that was first sold in liquid form in 2007. It is a toxicity category III product, is considered to have low mammalian toxicity but is irritating to the eyes and gastrointestinal tract, and might cause allergic skin reactions (5). No peer-reviewed in-vivo studies are available on triflumizole (6). Phosphoric acid is a toxicity category I product which, in pure form, can cause irreversible eye damage and skin burns. However, it is not likely to be responsible for illness because it is often used to achieve a neutral pH in pesticide mixtures. The pesticide mixture that the farmworkers were exposed to also contained mineral oil and boron, but these have low toxicity and are not thought to have contributed to illness onset.

The findings in this report are subject to at least three limitations. First, these workers were exposed to a mixture of several pesticides. It was not possible to determine if one active ingredient was responsible for the illnesses or if several were acting in concert. Second, symptoms of acute illnesses associated with pesticides are nonspecific and not pathognomonic, and diagnostic tests are not available to measure blood or urine levels of the pesticides involved in this event. Therefore false-positives might have been included as cases. Finally, samples for residue analysis were collected ≥3 days after the event. If the samples had been collected closer to the time of the event, more samples might have tested positive.

This event might have been prevented through better communication between managers of the cherry and pear orchards. Currently, only workers employed on the farm where the application is occurring must be notified about a pesticide application (Code of Federal Regulations (CFR) Title 40 Part 170.122). There is no Washington state or federal requirement to provide notification about pesticide applications to workers on a neighboring farm. There was anecdotal evidence to suggest that in the past, the managers of the two orchards involved in this event routinely and voluntarily shared information on upcoming pesticide applications to prevent pesticide exposures among workers in the neighboring orchard (Matt West, WSDA; personal communication; July 22, 2014). However, no such notification occurred in April 2014, possibly because both orchards experienced a recent turnover in management staff. Such a lack of notification to a neighboring farm is a frequent contributing factor to acute pesticide-related illness. Washington State Department of Health found that 31% of all acute pesticide-related illness cases identified among farmworkers during 2005–2012 involved exposure to off-target drift of pesticides that were applied to a neighboring farm (Joanne Prado, Washington State Department of Health; personal communication; August 18, 2014). In addition, a previous report documented lack of notification to a neighboring farm as a contributing factor in a cluster of acute pesticide-related illnesses in 2005 (7). At least one state health department (the California Department of Health Services) recommends that workers in nearby areas should be notified about scheduled pesticide applications, even when not required (7). Furthermore, although regulations prohibit applying agricultural pesticides in a manner that results in contact with workers or other persons (CFR Title 40 Part 170.210), the regulations do not explicitly state that applications must cease when the applicator observes workers or bystanders in neighboring, nontarget areas.

What is already known on this topic?

Off-target drift is the most common root cause for acute pesticide-related illness among farmworkers. Before an agricultural pesticide application is made, federal regulations require that workers employed on the farm where the application will be made be notified of the application. However, there is no requirement to notify the workers on adjacent farms of a pesticide application.

What is added by this report?

An off-target pesticide drift event occurred in April 2014, when pesticides applied to a pear orchard drifted over to a neighboring cherry orchard and quickly sickened all 20 farmworkers working in the cherry orchard. The vast majority reported neurologic, gastrointestinal, ocular, and respiratory symptoms. Six workers had moderate-severity illness, and the remaining 14 workers had low-severity illness. There are no previous reports in the literature of human illness caused by the three pesticides involved in this event (pyridaben, novaluron, and triflumizole).

What are the implications for public health practice?

This report highlights three potential occupational hazards in agriculture: off-target pesticide drift, toxicity of some recently marketed pesticides, and a gap in worker notification requirements. Incidents such as this could be prevented if farm managers planning pesticide applications notify their neighbors of their plans.

## Figures and Tables

**TABLE:** Signs and symptoms reported by 20 farmworkers exposed during a pesticide application — Douglas County, Washington, April 2014

Sign/Symptom*	No.	(%)
**Neurologic**	**20**	**(100)**
Headache	18	(90)
Paresthesias	14	(70)
Dizziness	12	(60)
Altered taste	10	(50)
Other†	6	(30)
**Gastrointestinal**	**19**	**(95)**
Nausea	15	(75)
Vomiting	10	(50)
Abdominal pain	9	(45)
Anorexia	3	(15)
**Eye**	**18**	**(90)**
Eye pain/irritation	16	(80)
Lacrimation	5	(25)
Conjunctivitis	3	(15)
**Respiratory**	**16**	**(80)**
Upper respiratory irritation	12	(60)
Dyspnea	10	(50)
Cough	4	(20)
Asthma exacerbation	2	(10)
**Dermatologic** §	**5**	**(25)**
**Cardiovascular** ¶	**2**	**(10)**

* The total number of signs/symptoms exceeds 20 because many persons had more than one sign or symptom.

† Other includes fatigue (one person), blurred vision (one), anxiety (one), fasciculations (one), and weakness (three).

§ Includes pruritis (four persons), rash (three), and redness (one).

¶ Includes elevated blood pressure (one person), palpitations (one).

## Footnotes

* Nexter miticide/insecticide; Gowan Company; U.S. Environmental Protection Agency (EPA) registration number 81880-4-10163. EPA toxicity category II. The toxicity of a pesticide is determined by EPA under guidance available from the Code of Federal Regulations 40 CFR 156.208(c) (2) (iii). Pesticides in category I are the most acutely toxic, and pesticides in category IV are the least.

† Rimon 0.83 EC insecticide; Makhteshim Agan of North America, Inc. EPA registration number 66222-35. EPA toxicity category II.

§ Procure 480SC agricultural fungicide; Chemtura Corporation. EPA registration number 400-518. EPA toxicity category III.

¶ Psylla is a major pear insect pest in North America. Additional information available at http://www.ipm.ucdavis.edu/pmg/r603301111.html.

** Hi Supreme spray oil; Independent Agribusiness Professionals. EPA registration number 71058-2. EPA toxicity category III. This was used as an insecticide.

†† Buffer-Ten; Monterey AgResources. California registration number 17545-50016. EPA toxicity category I.

§§ Standardized coding was used to determine severity of illness (information available at http://www.cdc.gov/niosh/topics/pesticides/pdfs/pest-sevindexv6.pdf). Low-severity cases usually resolve without treatment and cause minimal time lost from work or normal activities (<3 days). Moderate-severity cases are not life-threatening but require medical treatment and result in <6 days lost from work or normal activities.
